# CXCR6 by increasing retention of memory CD8^+^ T cells in the ovarian tumor microenvironment promotes immunosurveillance and control of ovarian cancer

**DOI:** 10.1136/jitc-2021-003329

**Published:** 2021-10-04

**Authors:** Ravikumar Muthuswamy, AJ Robert McGray, Sebastiano Battaglia, Wenjun He, Anthony Miliotto, Cheryl Eppolito, Junko Matsuzaki, Tsuji Takemasa, Richard Koya, Thinle Chodon, Brian D Lichty, Protul Shrikant, Kunle Odunsi

**Affiliations:** 1 Center For Immunotherapy, Roswell Park Comprehensive Cancer Center, Buffalo, New York, USA; 2 Biostatistics and Bioinformatics, Roswell Park Comprehensive Cancer Center, Buffalo, New York, USA; 3 University of Chicago Medicine Comprehensive Cancer Center, University of Chicago, Chicago, Illinois, USA; 4 Pathology and Molecular Medicine, McMaster University, Hamilton, Ontario, Canada; 5 Department of Microbiology and Immunology, University of Arizona, Tucson, Arizona, USA

**Keywords:** immunological memory, immunotherapy, immunotherapy, adoptive, lymphocytes, tumor-infiltrating, tumor microenvironment

## Abstract

**Purpose:**

Resident memory CD8 T cells, owing to their ability to reside and persist in peripheral tissues, impart adaptive sentinel activity and amplify local immune response, and have beneficial implications for tumor surveillance and control. The current study aimed to clarify the less known chemotactic mechanisms that govern the localization, retention, and residency of memory CD8 T cells in the ovarian tumor microenvironment.

**Experimental design:**

RNA and protein expressions of chemokine receptors in CD8^+^ resident memory T cells in human ovarian tumor-infiltrating CD8^+^ T cells and their association with survival were analyzed. The role of CXCR6 on antitumor T cells was investigated using prophylactic vaccine models in murine ovarian cancer.

**Results:**

Chemokine receptor profiling of CD8^+^CD103^+^ resident memory tumor-infiltrating lymphocytes in patients with ovarian cancer revealed high expression of CXCR6. Analysis of The Cancer Genome Atlas (TCGA) (ovarian cancer database revealed CXCR6 to be associated with CD103 and increased patient survival. Functional studies in mouse models of ovarian cancer revealed that CXCR6 is a marker of resident, but not circulatory, tumor-specific memory CD8^+^ T cells. CXCR6-deficient tumor-specific CD8^+^ T cells showed reduced retention in tumor tissues, leading to diminished resident memory responses and poor control of ovarian cancer.

**Conclusions:**

CXCR6, by promoting retention in tumor tissues, serves a critical role in resident memory T cell-mediated immunosurveillance and control of ovarian cancer. Future studies warrant exploiting CXCR6 to promote resident memory responses in cancers.

## Introduction

In patients with ovarian cancer, the density and location of CD8^+^ tumor-infiltrating lymphocytes (TILs) are critical for determining progression-free and overall survival.^1 2^ However, recent studies indicate that only ~10% of ovarian TILs are specific for shared antigens or mutated neoantigens.^3^ Moreover, the phenotype of tumor-specific T cells, rather than simply the sheer number of T cells, is a crucial parameter in determining effective cancer immunity.^3–5^ In this regard, eliciting T-cell responses with durable memory attributes in tumors can lead to better clinical outcomes in patients with cancer.^6 7^ Tissue-resident memory (Trm) T cells are a unique subset of memory T cells that lose their recirculatory potential and take up residency in peripheral tissues in areas of previous antigen encounter.^8–10^ This contrasts with central memory T cells (TCMs) that predominantly circulate within secondary lymphoid organs and effector memory T cells (TEMs), which recirculate mostly in peripheral tissues. Trm cells are considered as an amalgamation of TEM and TCM as they share features with both subsets, having effector molecules similar to TEM and renewal and longevity potential similar to TCM.^11^ Owing to their residency in peripheral tissues, they can act as adaptive sentinels capable of inducing rapid and robust antigen recall responses by eliciting systemic effector functions, without the requirement for priming in the lymph node.^12–14^ They are metabolically adept to persist and survive longer in peripheral tissues.^15^ Further, studies in mice and humans indicate their presence in tumors predicts enhanced tumor control and progression-free survival.^16–18^

Based on these attributes, Trm cells represent an important T-cell subset capable of generating robust antitumor immunity, and therapies like adoptive cell transfer (ACT) and immune checkpoint blockade will greatly benefit from improving their accumulation in tumors.^19 20^ However, little is known about the factors that govern Trm localization and retention in tumor tissues, one of the critical steps in the generation of Trm responses. Thus, identifying the factors and mechanisms that drive Trm localization and retention has profound therapeutic implications for promoting Trm response in tumors, as there are no strategies currently available to promote Trm response in patients with cancer.

Chemokines and chemokine receptors are implicated in the mobilization and localization of immune cells to peripheral tissues and the generation of memory response.^21–23^ While a role for CXCR6 in CD8^+^ T cell resident memory responses has been reported in infectious disease models,^24–26^ there are no studies that directly address if specific chemokine receptors facilitate resident memory response by driving localization or retention of CD8^+^ T cells in cancerous tissues. Our current study addresses this gap and identified CXCR6 as the predominant chemokine receptor expressed by CD8^+^ Trm cells in human ovarian cancer, and their presence in tumors is associated with increased survival. Using murine prophylactic vaccine models of ovarian cancer, we demonstrate that CXCR6 marks tumor-specific resident memory T cells. The deletion of CXCR6 in tumor-specific CD8^+^ T cells resulted in reduced retention in tumor tissues and increased CD8^+^ T-cell recirculation to the spleen, culminating in diminished resident memory response and reduced control of ovarian tumors. These findings indicate that CXCR6 is required for efficient generation of tumor-specific resident memory T-cell responses and could be therapeutically exploited for control of ovarian cancer.

## Materials and methods

### Human studies

Tissue samples were collected from patients undergoing primary tumor debulking surgery for ovarian cancer under a protocol approved by the institutional review board (protocol # I215512). For correlative analysis of immune markers and patient survival, a publicly available ovarian cancer TCGA Firehose legacy 2020 mRNA-seq database (n=307) was used. Out of this, only high-grade (stages IIIA–IV, n=280) patients were used for further analysis (online supplemental figure 1A). Correlation and survival analysis were done in either whole set (n=280) or in a subset of patients stratified for high CD8 levels (top 75 percentile, n=210, was used, while the bottom 25 percentile, n=70, was not included). For survival analysis with CXCR6, a comparison between the top and bottom 25 percentile was done either in whole or patients stratified for high CD8.

10.1136/jitc-2021-003329.supp1 Supplementary data

### Mice studies

Wild type (Wt.) and CXCR6 knockout (KO) C57BL/6 were purchased from The Jackson Laboratory (Bar Harbor, Maine, USA). OT1 PL RAGKO (Mice that expresses TCR for OVA_257-264_ peptide and has no endogenous CD4^+^, CD8^+^, T, and B cells due to KO in RAG gene) was a kind gift from Dr Shrikant. OT1 PL RAGKO mice were crossed with CXCR6KO to generate OT1 mice that were deficient for CXCR6. All the aforementioned crossing and expansion were done under breeding protocol 1145M, and experiments involving the mice were performed under protocol 1371M. All animals were maintained under pathogen-free conditions in the Laboratory Animal Shared Resource at Roswell Park Comprehensive Cancer Center (RPCCC). All animal experiments were carried out according to protocol guidelines reviewed and approved by the Institute Animal Care and Use Committee of RPCCC (Buffalo, New York).

### Adoptive cell transfer

OT1 CD8^+^ T cells from Wt. or the CXCR6KO mice were purified from spleens using EasySep Mouse CD8^+^ T Cell Isolation Kit (#19853; STEMCELL Technologies, Vancouver, Canada). Isolated CD90.1^+^ OT1 CD8^+^ T cells were injected into CD90.2^+^ C57BL/6 recipient mice at a concentration of 10^6^ cells/100 μL PBS through the retro-orbital route.

### Vaccination

Attenuated strain MG1 of Maraba virus^27 28^ expressing full-length ovalbumin (OVA) was manufactured at McMaster University, their titer determined, shipped on dry ice to RPCCC, and stored at −80°C before use. They were injected at 10^7^ Plaque Forming Units (PFU)() intraperitoneally for use as vaccine/adjuvant to activate OT1 T cells that were adoptively transferred 1 day before to C57BL/six mice.

### Tumor model

IE9-mp1, a derivative of widely used ID8 mouse ovarian surface epithelial cell line^29^ obtained by one passage in mice and genetically engineered to express OVA,^30^ was the tumor cell line used for in vivo mice study. Cell line was cultured in RPMI-1640 (10–040 CM)+10% fetal bovine serum (35–010-CV) in the presence of 1× penicillin/streptomycin (100 IU/100 μg/mL, 30–002 CI) at 37°C, 5% CO_2_ conditions. All reagents used for cell line culture were from Corning, Corning, New York. Mice were injected I.P. with 1×10^7^ cells of IE9-mp1. All the control and treated mice were euthanized when their abdominal circumference is ≈10 cm, which was considered as the experimental endpoint.

### Real-time qPCR analysis

RNA from tumor and cells were isolated by RNeasy kit (#74106, Qiagen,). cDNA conversion of isolated RNA was done by using iScript cDNA synthesis kit (#170889, BIO-RAD). cDNA was quantified by using predesigned KiCqStart SYBR Green primers (#KSPQ12012, Sigma Aldrich; sequences of the primers provided in online supplemental table 1) specific for the various human chemokine receptor genes and IQ SYBR green reagent (#170882, BIO-RAD) on CFX96 real-time PCR detection system (BIO-RAD). qPCR data were analyzed on CFX manager V.3.1 (BIO-RAD).

### Flow cytometry of human and mice samples

Human tumors were minced into small pieces, suspended in RPMI media, and homogenized into single-cell suspension using Miltenyi gentleMACS dissociater. The single-cell suspension was passed through a 100 µm cell strainer, washed, and spun on Ficoll to remove debris and dead cells. TILs processed previously were further processed for downstream analysis. For flow-based sorting of human Trm and non-Trm cells, processed TILs were stained for live/dead dye (Zombie Aqua fixable viability kit, #423 102 BioLegend) in PBS, followed by staining with BioLegend antibodies specific for CD3 (Pe-Cy7, HIT3a, #300316), CD8 (BV421, RPA-T8, #300106), CD103 (Alexa Flour 488, Ber-ACT8, #350208) and sorted on BD FACSAria II sorter into CD8^+^CD103^−^ (non-Trm) and CD8^+^CD103^+^ (Trm) cells and further processed for RNA analysis. Mice tumors were similarly processed, except for the Ficoll step, and were straightaway stained with flow antibodies. Human tumor samples were stained with live/dead dye in PBS, followed by staining with BioLegend antibodies specific for CD3, CD8, CD103, CCR5 (APC-cy7, J418F1, #359110), and with BD Biosciences antibodies specific for CXCR3 (PE, IC6, #560928), CXCR4 (APC,12G5, #560936), and CXCR6 (BV786, 13B1E5, #743602) in FACS buffer. Mice tissue samples were stained with live/dead dye, followed by antibodies specific for CD8 (FITC, 53–6.7, #553031), CD90.1 (PE, OX7, #554898), CD103 (A700, M290, #565529) from BD biosciences, CXCR6 (BV421, SAO51D1, #151109), CD44 (APC-Cy7, IM7, #103208), CD62L (BV786, MEL-14, #104440), CD69 (PE-Cy7, H1.2F3, #104512), and CXCR3 (BV650, CXCR3-173, #126531) from BioLegend. Stained samples were acquired on the BD LSRII flow cytometry system using BD FACSDiva software. Postrun analyses were done using the FCS Express V.7 Research Edition software (De Novo Software, Pasadena, California, USA).

### Confocal microscopy

Human or mouse tissue sections were marked on the edge with ImmEdge pen (H-4000, Vector Labs) and fixed in 4% Paraformaldehyde (PFA) for 15 min. Human and mouse tissues were blocked with 5% human or mouse serum, respectively, for 1 hour at room temperature (RT). This was followed by incubation with fluorophore-conjugated (1:100 dilution) or non-conjugated primary antibodies (1/500 dilution) for 3 hours at RT. Slides were washed four times in 1× PBS. To visualize antigens targeted with non-conjugated primary antibodies, fluorophore-conjugated secondary antibodies (1:1000 dilution, Cell Signaling Technology) were added along with nuclear dye (Hoechst 33422, #H1399; Thermo Fisher Scientific) for 30 min at RT, washed 4 four times in 1× PBS, and sections were mounted with coverslips in prolonged gold mounting medium. Tissues were analyzed in Leica SP8 DMRE spectral confocal microscope, and Image J software was used for postimage analysis. The full details of antibodies and secondary antibodies used for confocal staining of human and mouse tissues are given in online supplemental table 2).

### Cytotoxic T lymphocyte induced activated caspase3/7 assay

Wt. OT1 (5×10^5^) and CXCR6KO OT1 were incubated by itself or with 5×10^4^ of OVA expressing IE9-mp1 target cell line or OVA non-expressing ID8 cell in the presence of 4 µM CellEvent Caspase-3/7 Green Detection Reagent (#C10423, Thermo Fisher Scientific) in triplicate for 24 hours. Caspase-3/7 fluorescence is measured on Biotek Synergy HT microplate reader at emission 528 nM. Images of the wells with each condition were taken on ZOE fluorescent cell imager (BIO-RAD).

### Statistical analysis

All statistical analyses involved in the study were performed using GraphPad Prism 8 software. The type of statistic used is described in the figure legends, respectively. For all analyses, p<0.05 was considered statistically significant; *, **, ***, and **** indicate p<0.05, p<0.01, p<0.001 and p<0.0001, respectively. All data were expressed as mean±SEM except in some cases, where box and whisker plots were used. In the box and whisker plots, the horizontal line represents the median and the whiskers connect the highest and lowest observations. Log-rank (Mantel-Cox) test was used to calculate the mice survival.

## Results

### Chemokine receptor CXCR6 is highly expressed on human tumor-resident memory CD8^+^ T cells

**Figure 1:**
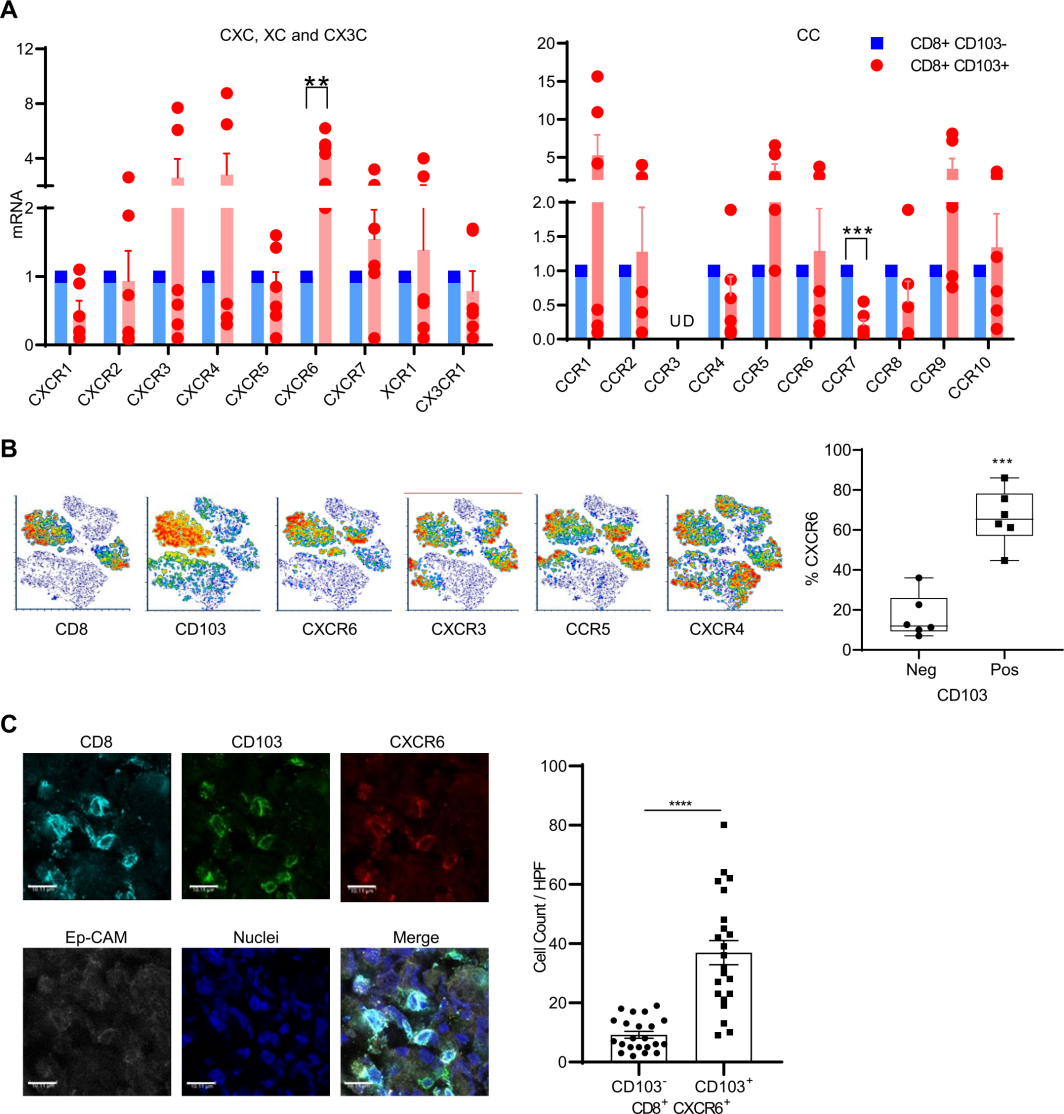
Chemokine receptor CXCR6 is a valid marker of human tumor resident CD8^+^ T cells. (A) CD8^+^ TILs from patients with ovarian cancer (n=6) were flow-sorted into CD103^+^ (Trm) and CD103^−^ (non-Trm) subsets and real-time-Quantitative Polymerase chain reaction (RT-qPCR) was used to profile the expression of CXC, XC and CX3C (left), and CC (right) chemokine receptors. mRNA levels were normalized to HPRT1 and chemokine mRNA levels in the CD103^+^ subset were expressed as fold change over the CD103^−^ subset. Data presented as mean±SEM. (B) CD8^+^ TILs in human ovarian cancer were flow stained for CD103 and chemokine receptors and presented in the left panel as T-distributed Stochastic Neighbor Embedding (TSNE) plot from one representative patient, and in the right panel as box and whisker plots of %CXCR6 in CD103^−^ and CD103^+^ CD8^+^ TILs from six patients with ovarian cancer. (C) Left: images from confocal staining for CD8^+^CD103^+^CXCR6^+^ Trm cells in ovarian cancer (scale bars are at 10 µm); right: bar graphs represent 22 high-power field (HPF) CD103 positive and negative cell counts in CD8^+^CXCR6^+^ TILs from five patient tumors. (A–C) Paired two-tailed t-test was used. **P<0.01, ***P<0.001, ****P<0.0001. OVA, ovalbumin; TIL, tumor-infiltrating lymphocyte; Trm, tissue-resident memory; UD, undetectable.

To characterize the potential chemokine/chemokine receptor axes involved in the localization of Trm CD8^+^ T cells in the ovarian cancer tumor microenvironment (TME), we profiled the expression of the known chemokine receptors in Trm (CD3^+^CD8^+^CD103^+^) versus non-Trm (CD3^+^CD8^+^CD103^−^) cells obtained from patients with ovarian cancer tumors using Realtime- quantitative polymerase chain reaction (RT-QPCR) and flow cytometry. We used CD103 for identifying Trm cells, as it is considered a canonical marker for Trm cells and predicts favorable outcomes in patients with ovarian cancer.^31 32^ While mRNA expression of most chemokine receptors demonstrated no clear differences between Trm and non-Trm, Trm cells consistently showed significant upregulation of CXCR6 mRNA compared with non-Trm cells (figure 1A). CCR7 mRNA showed a reverse trend with more expression in non-Trm cells (figure 1A). Flow cytometry analysis further confirmed the high expression of CXCR6 protein in CD8^+^CD103^+^ Trm cells (figure 1B), in agreement with previous reports.^33^ Although CXCR3 was high in CD103^+^ Trm cells, no significant difference was noted in its expression when compared with non-Trm CD8^+^ T cells. Analysis with confocal microscopy further substantiated the coexpression of CXCR6 and CD103 on CD8^+^ TILs (figure 1C).

### TCGA analysis reveals a positive correlation of CXCR6 with CD103 and survival of patients with ovarian cancer

**Figure 2:**
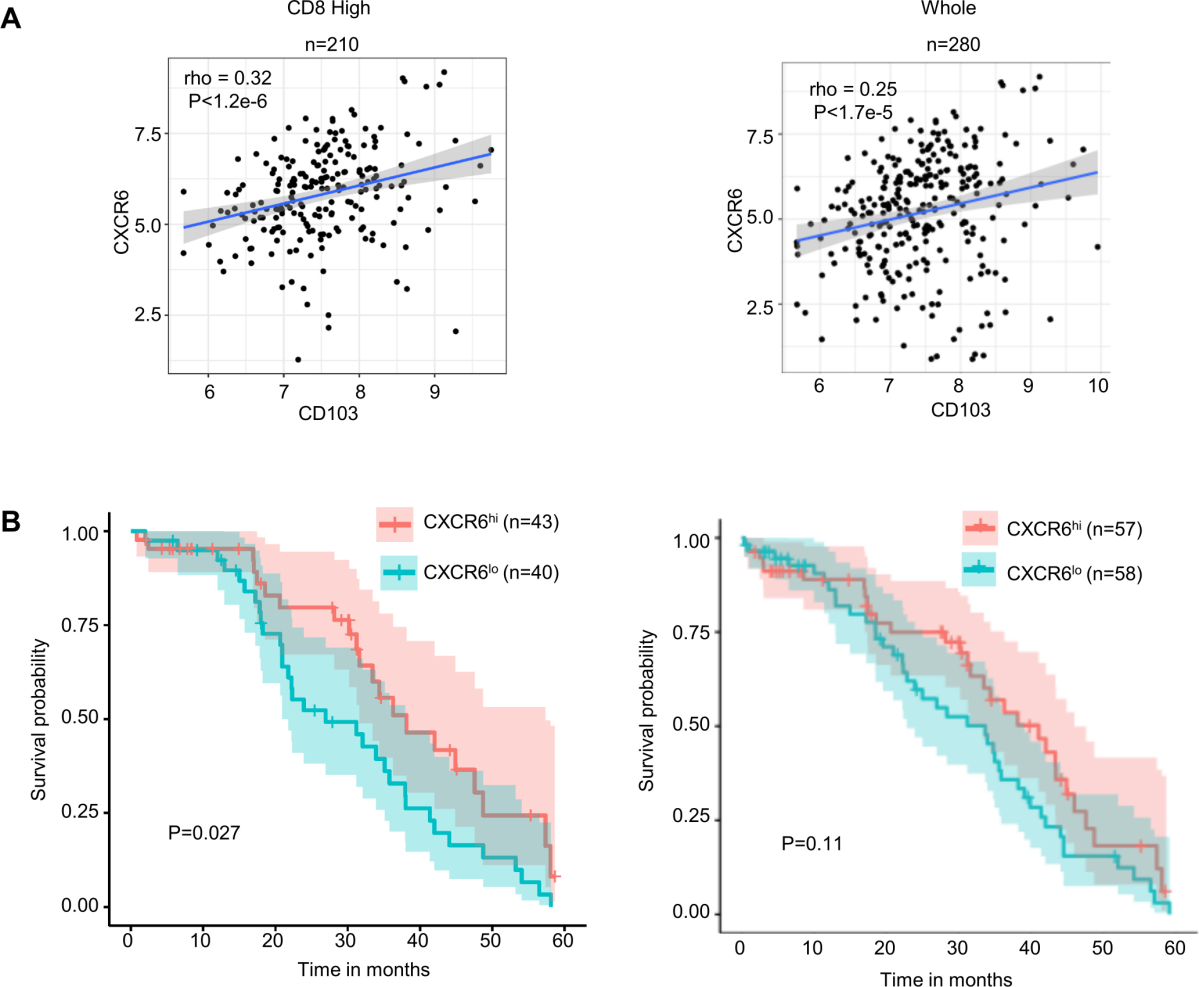
CXCR6 shows a high correlation with CD103 and protection against ovarian cancer based on ovarian TCGA database analysis. (A) Spearman’s rank correlation between CXCR6 and CD103 markers was done in the CD8 high (left, n=210) or the whole set (n=280) of high-grade TCGA ovarian patient database. Analysis of association of CXCR6 (B) markers with survival in the CD8 high (left) or the whole set (right) of TCGA database of patients with high-grade ovarian cancer.

Analysis of the whole database of patients with high-grade serous ovarian cancer (n=280) or patients stratified for high CD8 (n=210) showed a significant correlation between CD103 and CXCR6 mRNA markers (figure 2A). Details of TCGA database stratification are provided in online supplemental figure 1A-C). Furthermore, survival analysis using TCGA data revealed that the CXCR6 marker to be positively associated with increased survival in CD8 high but not in the whole dataset (figure 2B).

### Mice treated with adoptive transfer of OT1 and vaccination with OVA-expressing Maraba virus (Mrb-OVA)-treated mice display higher survival

**Figure 3:**
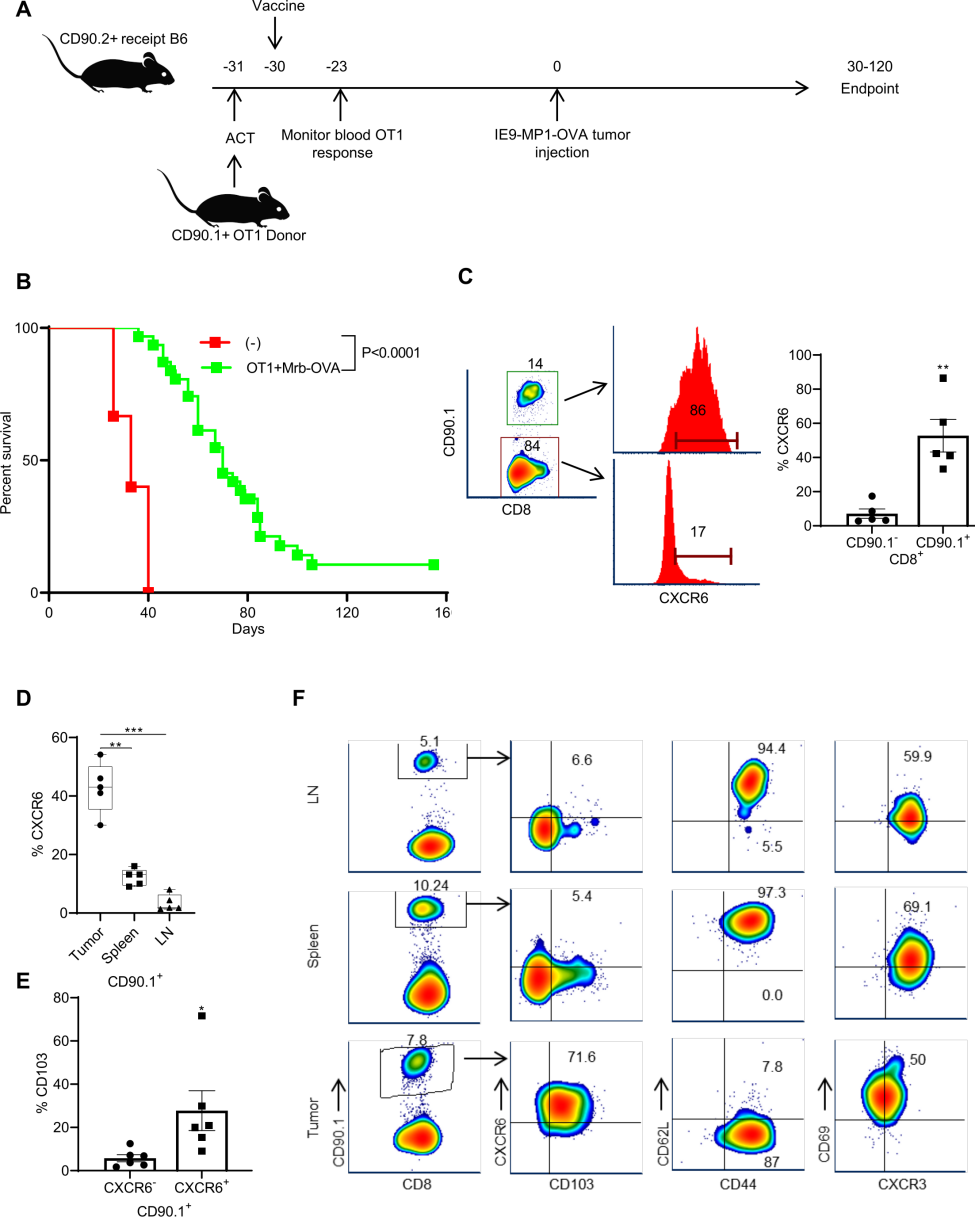
OT1+Mrb OVA-treated mice display higher survival and CXCR6 defines tumor-specific resident memory CD8^+^ T cells in murine ovarian cancer models. (A) The experimental schema for adoptive cell transfer of OT1 cells in conjunction with Mrb-OVA vaccination is shown. (B) Comparison of survival between mice treated with Phosphate Buffered Saline (PBS) and with OT1+Mrb OVA combination. Mice are n=31 for OT1+Mrb OVA and n=15 for control. Log-rank (Mantel-Cox) test was used to calculate the mice survival, and median survival is 70 for OT1+Mrb OVA and 33 for control groups. (C). CXCR6 expression in non-tumor-specific (CD90.1^−^) and tumor-specific (CD90.1^+^) CD8^+^ T cells in tumors of mice treated with OT1+Mrb OVA. (D) Comparison of CXCR6 expression in CD90.1^+^ tumor-specific CD8^+^ T cells present in tumor, spleen, and lymph nodes (n=5) and shown as box and whisker plots. (E) Bar graphs of %CD103 in CXCR6^−^ and CXCR6^+^ subsets of OT1 CD8^+^ T cells in tumor tissues of OT1+Mrb OVA-treated mice (n=6). (F) Flow cytometry density plots of CD103 and other memory marker expression in CD90.1 gated CD8^+^ T cells in lymph nodes, spleen, and tumors of one representative OT1+Mrb OVA-treated mice. (C–E) A two-tailed paired t-test was used to analyze data. *P<0.05, **P<0.01, ***P<0.001. Data in bar graphs (C, E) are mean±SEM. Mrb-OVA, OVA-expressing Maraba virus; OVA, ovalbumin.

To directly test whether CXCR6 plays a functional role in the localization and efficacy of tumor-resident memory responses, we developed a prophylactic vaccination model using a mouse intraperitoneal ovarian cancer model as previously described.^34 35^ We used the prophylactic model instead of the therapeutic setting for the following reasons: (1) the continued presence of tumors may promote more of the effector rather than memory responses; (2) response to the tumor may not necessarily be derived from memory CD8^+^ T cells; and (3) the OVA-expressing IE9-mp1 tumor model is highly aggressive, and the cure rate is low,^35^ which makes priming and challenge with the tumor difficult. This model (figure 3A) uses ACT of naïve OT1 CD8^+^ T cells into C57BL/6 recipient, followed by priming with Mrb-OVA vaccination to activate OT1 cells. We chose OT1 T cells for adoptive transfer to allow for careful monitoring and tracking of T-cell responses as shown in previous studies.^13 36^ Mrb-OVA was used as the vaccine as it was shown to induce robust antitumor CD8^+^ T-cell responses in our previous study.^35^ Tumor challenge was done 31–30 days after adoptive transfer of OT1 and vaccination with Mrb-OVA. This is to give enough time for the vaccine-induced effector response to subside and allow memory generation. This increases the probability that antitumor immunity following tumor challenge would come from vaccine-induced memory cells. In this model, the OT1+Mrb OVA combination provides better tumor control than individual treatments with either OT1 or Mrb-OVA (online supplemental figure 2A-C). As shown in figure 3B, the combination of ACT and vaccination with Mrb-OVA improved tumor protection compared with no treatment as indicated by a median survival of 70 days vs 33 days.

### CXCR6 is highly expressed in antigen-specific CD8^+^ T cells that reside in the tumor, but not those in circulation

Analysis of endpoint tumors from mice that were treated with OT1+Mrb OVA showed that CXCR6 expression was seen predominantly in tumor-specific CD90.1^+^ OT1 (figure 3C), but less frequently in CD90.1^−^ CD8^+^ T cells. Similarly, analysis of endpoint tumors in Mrb-OVA alone treated mice (online supplemental figure 2) revealed that the endogenous tumor-specific CD8^+^ T cells (marked by OVA tetramer staining) showed high CXCR6 expression compared with non-specific (OVA tetramer negative) T cells (online supplemental figure 3A). When CXCR6 expression was compared between OT1 CD8^+^ T cells in tumor, spleen, and lymph node tissues of OT1+Mrb OVA-treated mice that had reached endpoint, only tumor-infiltrating OT1 T cells showed high CXCR6 expression (figure 3D and F). Analysis of CD103 in CD90.1^+^ TILs revealed high expression by CXCR6^+^ cells, rather than in the CXCR6^−^ subset (figure 3E and F).

CD44 and CD62L marker-based stratification showed that CD90.1^+^ TILs were predominantly of CD44^+^CD62L^−^ effector memory phenotype, whereas those in lymph nodes and spleen were more of central memory type (figure 3F). The observation that Trm cells resembled more of the effector/effector memory than the central memory phenotype is in agreement with previous observations.^17 37 38^ Comparison of OT1 CD8^+^ T cells prior to transfer (top), 7 days post-transfer in recipient blood (middle) and in endpoint tumors (lower) revealed again that CXCR6 is acquired by T cells only in tumors and marks CD44^+^CD62L^−^CD103^+^ tumor-resident memory cells, but not circulatory cells (online supplemental figure 3B).

Analysis of additional Trm markers, namely, CD69 and CXCR3, revealed that they were found in both circulatory and resident memory CD90.1^+^CD8^+^ T cells (figure 3F and online supplemental figure 3B). This discrepancy between the current study and other studies concerning CD69 as a Trm marker can be attributed to the different tissue microenvironment as suggested by Walsh *et al*.^39^ Expression of CXCR3 on both resident memory and circulatory memory cells in murine studies further supported our observations in human Trm. Consistent expression of CXCR3 on both subsets and selective late expression of CXCR6 on resident memory cells suggest that while CXCR3 may direct default peripheral migration of both memory subsets to the tumor, CXCR6 selectively promotes tissue residency of memory cells. The aforementioned observations strongly attest to the validity of CXCR6 as a Trm marker with functional implications in resident memory response to ovarian tumors.

Cells negative for CD45 and EpCAM contribute more to CXCL16 expression than hematopoietic or epithelial cells to help Trm cells localize within the ovarian TME.

**Figure 4:**
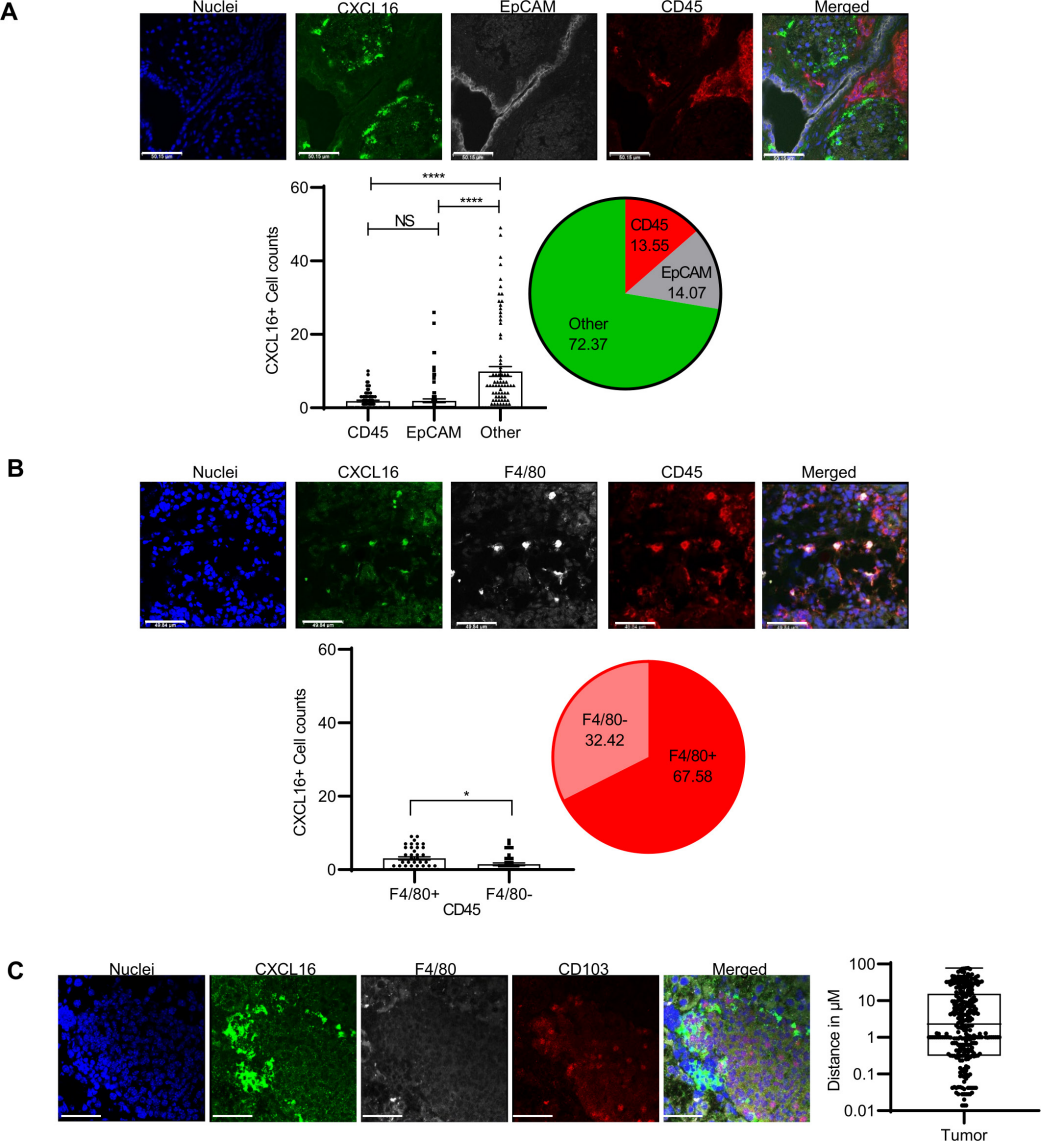
Cells negative for CD45 and EpCAM contribute more to CXCL16 expression to help localize Trm cells in mice ovarian tumor microenvironment. Endpoint tumors from OT1+Mrb OVA-treated mice were stained for nuclei (blue), CXCL16 (green), along with (A) CD45 (red) and EpCAM (gray) or with (B) CD45 (red) and F4/80 (gray) or with (C) F4/80 (gray) and CD103 (red). (A) Bar graphs represent the total cumulative of 84 high-power fields (×63 magnification) cell counts from 16 tumors, whereas for (B), it is the total cumulative of 40 high-power fields (×63 magnification) cell counts from 15 tumors. The pie diagram represents % contributions from each cell type calculated based on cell counts. (C) A total of 296 distance measurements between cells positive for CD103 and CXCL16 from five mice tumors. Scale bars are at 50 µm (A–C). NS, not significant, * P<0.05, **** P<0.0001; Trm, tissue-resident memory.

As CXCL16 is the main identified ligand for CXCR6,^40^ we wanted to identify the cells that produce the CXCL16 in the murine ovarian TME. We stained tissues from endpoint tumors from OT1+Mrb-OVA-treated mice with antibodies for mouse CXCL16 and EpCAM (tumor cell marker) and CD45 (immune cells). Confocal microscopy analysis (figure 4A) revealed multiple sources of CXCL16, as revealed by CXCL16 (Green) staining in EpCAM^+^ (gray), CD45^+^ (red), and in the cells that were negative for EpCAM and CD45. However, quantitative analysis revealed that the latter EpCAM^−^CD45^−^ cells were the dominant producers of CXCL16, as they contributed to 72% of CXCL16 expression compared with EpCAM^+^ and CD45^+^ cells that contributed 13% and 14%, respectively (figure 4A). Additional analysis among CD45^+^ cells revealed that F4/80^+^ cells were the major producers of CXCL16, as they contributed about 67% to CXCL16 expression within immune cells (figure 4B). To further substantiate the CXCL16 role in Trm localization, we stained for F4/80, CD103, and CXCL16 markers in endpoint tumor tissues and analyzed them using confocal immunofluorescence microscopy. The analysis revealed that most CD103^+^ T cells were found proximal to CXCL16-positive cells (figure 4C), with most of them within 20 µm distance. The aforementioned observations suggest that multiple sources of cells express CXCL16 in the ovarian TME to support the local accumulation of CXCR6^+^ Trm cells.

### KO of CXCR6 in tumor-specific CD8^+^ T cells enhances circulatory but reduces resident memory response in tumors, leading to diminished protection against ovarian cancer

**Figure 5:**
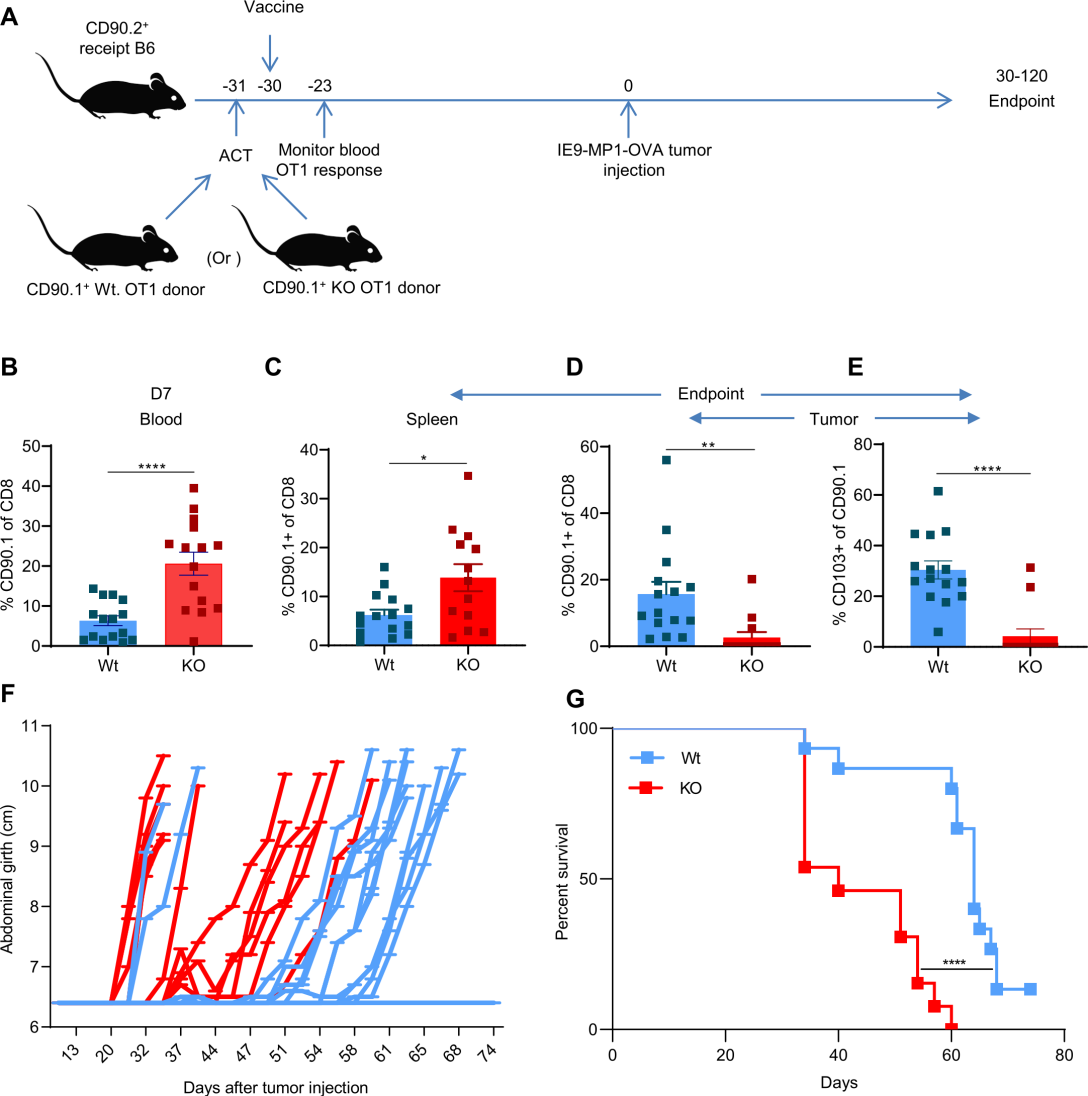
CXCR6KO enhances T-cell response in blood but conversely lowers it in tumor tissues and weakens ovarian tumor control in recipient mice. (A) The experimental schema for ACT of Wt. or CXCR6KO (KO) OT1 cells in conjunction with Mrb-OVA vaccination in B6 recipient mice is shown. (B) %CD90.1^+^ T cells in day 7 postvaccination blood of B6 recipient mice that received Wt. or KO OT1 cells, n=15 for each. Comparison of %CD90.1^+^ T cells in the spleen (C), tumor (D), CD103^+^ T cells in the tumor (E) at the endpoint, tumor progression (F), and survival (G) between the two groups of B6 mice that received ACT of either Wt. (n=15) or KO (n=13) OT1 cells+Mrb OVA vaccination. Non-paired two-tailed t-test was used to analyze data in (B–E), and data are presented as mean±SEM log-rank (Mantel-Cox) test was used to calculate the survival between mice belonging to Wt. and KO groups (G) with the median survival of 64 and 40, respectively. *P<0.05, **P<0.01, ****P<0.0001. ACT, adoptive cell transfer; KO, knockout; Mrb-OVA, OVA-expressing Maraba virus; Wt., wild type.

To determine if CXCR6 plays a critical functional role in Trm generation and tumor immunity, CXCR6KO OT1 mice were generated by crossing OT1 PL RAGKO mice with CXCR6KO mice. In vitro testing confirmed no clear phenotypical or functional differences between Wt. and CXCR6KO OT1 T cells, as no differences were noted in marker expression (Online supplemental figure 4A) or their lytic capacity against IE9-mp1 target cells (online supplemental figure 4B). Wt. or CXCR6KO (KO) CD90.1^+^ OT1 cells were adoptively transferred into C57BL/6 recipient mice (CD90.2^+^) on day −31, followed by vaccination with Mrb-OVA at day −30 and tumor implantation on day 0 (figure 5A). Blood analysis on day −23 (7 days after ACT+vaccination) revealed the C57BL/6 recipients that received the adoptive transfer of KO T cells showed significantly higher circulatory T-cell response than those that received Wt. T cells (figure 5B). This was backed by similar findings in the spleen of C57BL/6 recipient mice that received KO T cells, as they again showed a higher percentage of CD90.1^+^ T cells compared with Wt. recipients (figure 5C and online supplemental figure 5). In contrast, quantification of TILs in endpoint tumors revealed that C57BL/6 recipients of CD8^+^ T cells from KO mice had few or no detectable CD90.1^+^ TILs compared with that received from Wt. mice, where CD90.1^+^ TILs were consistently detected (figure 5D). This was also true for CD103^+^ TILs, as they were also at low frequency or undetectable in recipient mice that received KO compared with Wt. OT1 (figure 5E). This difference in transferred T-cell persistence in tumors was reflected by differences in tumor progression (figure 5F) and survival (figure 5G), as C57BL/6 recipients of Wt. CD8^+^ T cells controlled tumors significantly better than KO T-cell recipients. Together, the observations confirm that loss of CXCR6 reduces resident memory responses and the associated protection against ovarian cancer.

## Discussion

The underpinning chemotactic mechanisms guiding Trm localization and retention in the TME and preventing their emigration have not been clearly defined. Several mechanisms including induction by specific subsets of DC; expression of specific transcriptional regulators HOBIT, BLIMP, or Runx3; acquisition of integrins; exposure to homeostatic cytokines and inflammatory signals; and improved metabolic fitness have all been implicated in Trm generation and persistence.^15 37 41–44^ However, most studies have used infectious models, and the relevance of these factors and associated mechanisms for the generation of Trm responses in cancer is less understood. Given the beneficial role of Trm in tumor control and the increasing need to develop strategies to promote Trm response in tumors, understanding how Trm response is generated and maintained in tumors is warranted. Specifically, identifying the chemotactic mechanisms that drive Trm response to tumors will greatly help in the design of immunotherapeutic strategies to promote Trm in patients’ tumors, potentially leading to improved therapeutic efficacy and clinical outcome. Our studies in both humans and mice strongly indicate that CXCR6 is highly expressed on Trm cells, play a critical role in their localization within tumor tissues, and impart increased protection against ovarian cancer. While a role for CXCR6 in CD8^+^ Trm responses using infectious disease models^24–26^ has been reported, to date, there are no reports on the functional role of CXCR6 in mediating Trm responses to tumors. This is critical as most mechanisms underpinning immunity against infectious agents versus tumor challenge are distinct. To our knowledge, this is the first study to demonstrate the indispensable requirement for CXCR6 in Trm responses and the role in mediating ovarian cancer immunity.

Several lines of evidence from our studies support the requirement for CXCR6 in mediating Trm responses. Our chemokine receptor profiling studies of human ovarian cancer TILs using qPCR, flow cytometry, and confocal microscopy all revealed high expression of CXCR6 on CD8^+^ CD103^+^ Trm cells. Analysis of TCGA database of patients with ovarian cancer further corroborated this strong association between CXCR6 and CD103. Further, CXCR6 was found associated with increased survival only with patients with CD8-high ovarian cancer.

To corroborate the human findings of preferential expression of CXCR6 on ovarian tumor-infiltrating Trm cells, we used prophylactic vaccine models in murine ovarian tumors. The advantage of using the prophylactic model over the therapeutic model is that it increases the probability of tumor responses to be derived from memory CD8^+^ T cells. This is not possible in the therapeutic model, where the constant presence of tumor mitigates memory formation or makes it dysfunctional. Our results from mice extend the human observations on CXCR6 and Trm cells. Additionally, it suggests that chemokine receptor CXCR6 is not involved in T-cell trafficking to tumors, as that is served by CXCR3, which shows high expression on both resident and circulatory memory T cells. This unbiased expression of CXCR3 on both memory subsets is also seen in human samples. In contrast, CXCR6 serves as a retention factor to keep T cells in ovarian peritoneal metastatic sites, increasing their likelihood of becoming resident memory cells. This is supported by the fact that CXCR6 expression is acquired late and occurs in situ in the tumor, and CXCR6 is highly expressed in tumor-specific T cells that are resident but not by those in circulation. The strong association of CXCR6 with CD103 (a marker of tissue residency) in both human and mouse studies also attests to the retention role. Further, the observation of proximity of CD103^+^ T cells to cells that express CXCL16 (the primary chemokine ligand for CXCR6) serves as additional evidence for the role of CXCR6 in Trm cell localization in ovarian tumor tissues. Characterization of cells that produce CXCL16 and help localize CXCR6^+^CD103^+^ within the ovarian TME revealed that the majority of these CXCL16-producing cells were negative for CD45 and EpCAM. This finding warrants further characterization of these cells in the future. KO of CXCR6 in tumor-specific T cells enhanced their responses in blood and spleen. However, they showed a reduced response in the tumor as evidenced by low frequencies of CD90.1^+^ TILs and, more specifically, fewer numbers of CD103^+^ resident memory cells in the tumor. This culminated in poor control of tumors by KO T cells in recipient mice. Although we observed some tumor control even in recipient mice of KO T cells, this might be due to some contributory responses from circulatory memory cells, as reported previously.^45^

Based on the aforementioned results, we hypothesize that DC/tumor antigen-activated T cells express CXCR3, driving T-cell infiltration into the tumor or peripheral tissues. Though the current study did not verify CXCR3 role in peripheral migration using CXCR3KO studies, previous studies support this fact.^46 47^ Once in the tumor, in situ acquisition of CXCR6 dictates whether T cells will remain localized to the tumor or emigrate from the ovarian peritoneal tissue microenvironment. CXCR6 on tumor-specific T cells then engages with CXCL16 derived from the TME and facilitates T-cell retention. The role of CXCR6 in T-cell retention is supported by the previous demonstration that the unique DRF motif in CXCR6 makes it more suited for adhesion than chemotaxis to ligand CXCL16,^48^ which exists in both transmembrane and soluble forms.^49^ We envisage that CXCR6-mediated retention may increase the probability of T-cell exposure to cytokines like interleukin-15, Transforming Growth Factor-beta(TGF-β), and other influences in tissue milieu that drive Trm development.^36 37 50^

In conclusion, the present study implicates CXCR6 as a critical regulator of residency and persistence of memory CD8^+^ T-cell responses in ovarian TME, thereby increasing enhanced immunosurveillance and control of ovarian cancer. The study provides support for the development of CXCR6/CXCL16-targeted therapeutic approaches to enhance antitumor Trm retention within the TME for improved treatment outcomes. Such approaches could include nanoparticle or gene therapy delivery of CXCR6 and CXCL16 into T cells or the TME, respectively.

## Data Availability

Data are available upon reasonable request.
