# Supplementary material for: CXCR6 by increasing retention of memory CD8+ T cells in the ovarian tumor microenvironment promotes immunosurveillance and control of ovarian cancer
Source: J Immunother Cancer. 2021 Oct 4;9(10):e003329. doi: 10.1136/jitc-2021-003329 (PMC8491420; doi:10.1136/jitc-2021-003329)
Supplement: Supplementary data [file jitc-2021-003329supp001.pdf]

Supplementary Table-1. Primers for SYBR based real-time QPCR analysis of Human genes

Gene Name		Sequence 5'-3'	# CAT	Company
CCR1	Forward	CCTTGGAACCAGAGAGAAG	KSPQ12012	Sigma-Aldrich
	Reverse	AATACCAAGGAGTACAGAGG	"	"
CCR2	Forward	AAGCCTTTTTCACATAGCTC	"	"
	Reverse	CTTTCACATTCTTTCCTGGTC	"	"
CCR3	Forward	CACTGCTGAGTTGTATTGG	"	"
	Reverse	GCTCTGGTATCAGCTTTTTC	"	"
CCR4	Forward	GCTTTCAGAAAAGCAAGC	"	"
	Reverse	TATTCTGTGTAGTGGGATGAG	"	"
CCR5	Forward	TGCTGTTTCTTTTGAAGGAG	"	"
	Reverse	TATGCTGGTGAACAGAAATG	"	"
CCR6	Forward	CTCAATAAAGAAGGAGCTGTC	"	"
	Reverse	GAACAAAGGCTGTCACTAAG	"	"
CCR7	Forward	AATGATGGAGTACATGATAGGG	"	"
	Reverse	CAGACAAGCAAAACAAAGTG	"	"
CCR8	Forward	GAACAAAGGCTGTCACTAAG	"	"
	Reverse	GTTTCCCAGAAGACTGAATAC	"	"
CCR9	Forward	GACTAACACAAGCCCTATTC	"	"
	Reverse	CACAGTAGAAGTCAGTGAAG	"	"
CCR10	Forward	CTGCGAATCTAGAGGAGG	"	"
	Reverse	CACAGAGGTAGTCCCTTTAG	"	"
XCR1	Forward	AGAAACACCAGGCAGTATAG	"	"
	Reverse	GACTAACACAAGCCCTATTC	"	"
CXCR1	Forward	TTAAGTCACTCTGATCTCTGAC	"	"
	Reverse	TGGTTTGATCTAACTGAAGC	"	"
CXCR2	Forward	CCAGTCAGGATTTAAGTTTACC	"	"
	Reverse	GTTGATTTCCAGGGATTCTG	"	"
CXCR3	Forward	GTCCTTGAGGTGAGTGAC	"	"
	Reverse	TCTCCATAGTCATAGGAAGAG	"	"
CXCR4	Forward	AACTTCAGTTTGTTGGCTG	"	"
	Reverse	GTGTATATACTGATCCCCTCC	"	"
CXCR5	Forward	AGTATCCTCATTTGGGGTAG	"	"
	Reverse	GCATTGGATGATTAGGATGG	"	"
CXCR6	Forward	GGTGTTCATCAGAACAGAC	"	"
	Reverse	GAAAGACCTTGCTGAACTG	"	"

CXCR7	Forward	GATGTGGGTTACAAAGCTG	"	"
	Reverse	AATCAAATGACCTCCGGG	"	"
CX3CR1	Forward	AAATACCCCATCATTCATGC	"	"
	Reverse	TTGTTCCAAACGTTTCTAGG	"	"
HPRT1	Forward	ATAAGCCAGACTTTGTTGG	"	"
	Reverse	ATAGGACTCCAGATGTTTCC	"	"

 BMJ Publishing Group Limited (BMJ) disclaims all liability and responsibility arising from any reliance placed on this supplemental material which has been supplied by the author(s)

Supplementary table 2A. Details of primary and secondary antibodies used for confocal staining of human tumor tissues.

**Primary antibodies**	**Target Antigen**	**Antibody clone**	**Isotype**	**Flourophore**	**Cat#**	**Vendor**
	CD103	EPR4166(2)	Rabbit Monoclonal	None	ab129202	Abcam
	CXCR6	K041E5	Mouse IgG2a	None	356002	BioLegend
	EPCAM	9C4	Mouse IgG2b	APC/Fire-750	324233	BioLegend
	CD8	YTC182.20	Rat IgG2b	None	MCA351G	Bio-Rad
**Secondary antibodies**	**Target Antigen**	**Antibody clone**	**Isotype**	**Flourophore**	**Cat#**	**Vendor**
	Rabbit IgG	Polyclonal	Goat IgG	Alexa-488	4412s	Cell signaling Technology
	Rat IgG	Polyclonal	Goat IgG	Alexa-555	4417s	Cell Signaling Technology
	Mouse IgG	Polyclonal	Goat IgG	Alexa-647	4410s	Cell signaling technology

Supplementary table 2B. Details of primary and secondary antibodies used for confocal staining of mouse tumor tissues.

	**Target Antigen**	**Antibody clone**	**Isotype**	**Fluorophore**	**Cat#**	**Vendor**
**Primary antibodies**	CXCL16	Polyclonal	Rabbit	None	119350	Abcam
	CD103	M290	Rat IgG2a	A700	565529	BD Biosciences
	EP-CAM	G8.8	Rat IgG2a	Alexa594	118222	BioLegend
	CD45	30-F11	Rat IgG2b	Alexa647	103124	BioLegend
	F4/80	BM8	Rat IgG2a	Alexa594	123140	BioLegend
**Secondary antibodies**	**Target Antigen**	**Antibody clone**	**Isotype**	**Flourophore**	**Cat#**	**Vendor**
	Rabbit IgG	Polyclonal	Goat IgG	Alexa-488	4412s	Cell Signaling Technology

A

Stage	No of Patients
III A	7
III B	14
III C	221
IV	38
Total	280

B

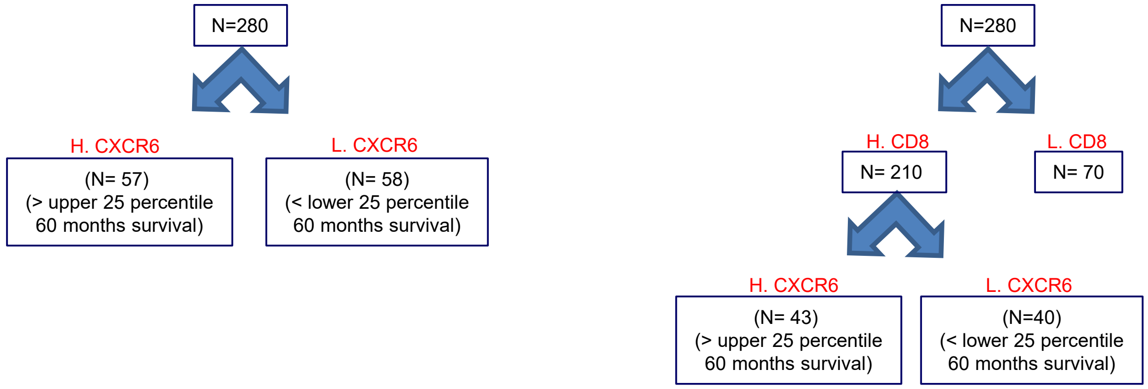

Supplementary figure 1. Details of high Grade TCGA patient database and stratification. (A) Info on the stages and no patients in each stage for the TCGA database of Ovarian cancer patients used in the current study. Strategy of sample stratification of TCGA database for to analyze **(B)** correlation and survival with markers CXCR6.

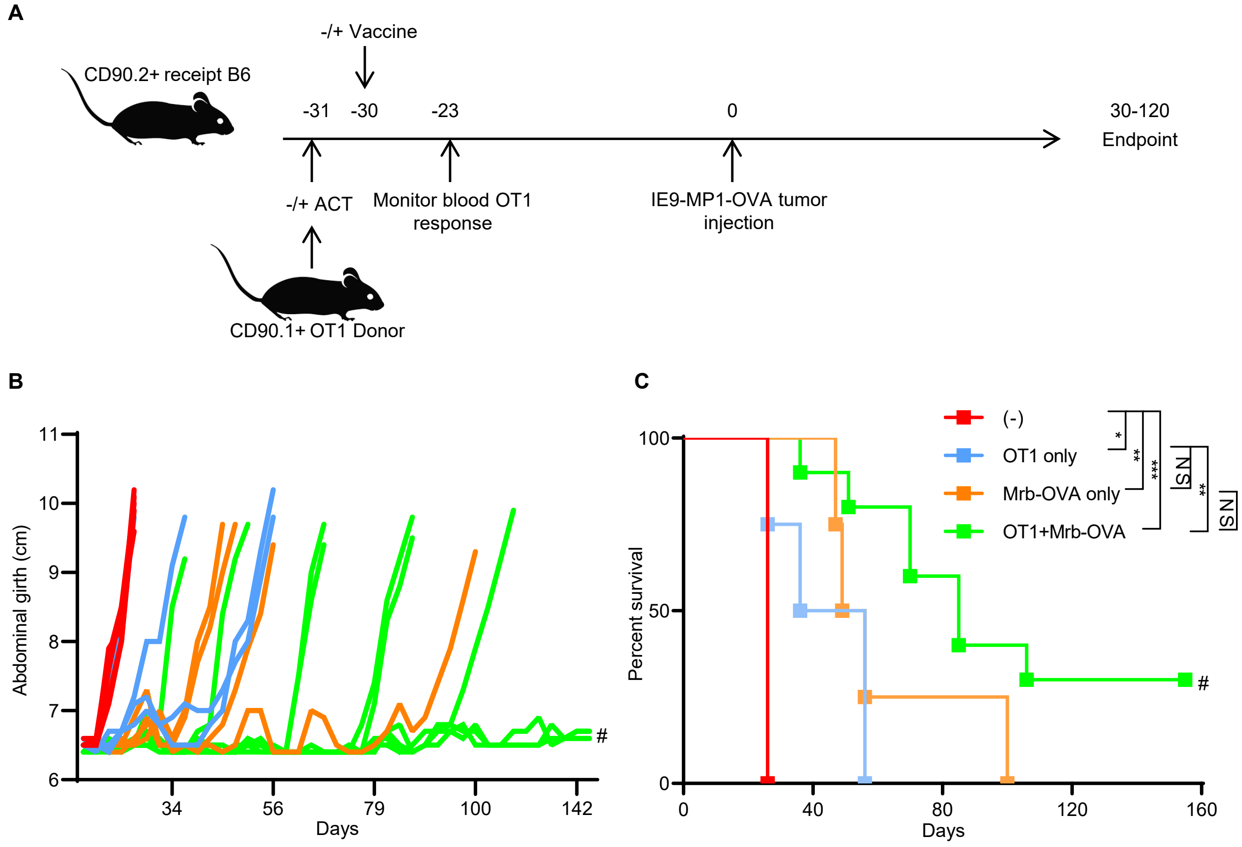

Supplementary figure 2. Treatment with combination of adoptive transfer OT1 and vaccination with Mrb-OVA offers better protection than single treatments. (A) Experimental schema of the prophylactic model used to assess test therapeutic efficacy of individual or combination treatments with OT1 T cells and Mrb-OVA vaccine. Tumor progression **(B)** and survival **(C)** of mice treated with individual or combination of OT1 T cells and Mrb-OVA vaccine.

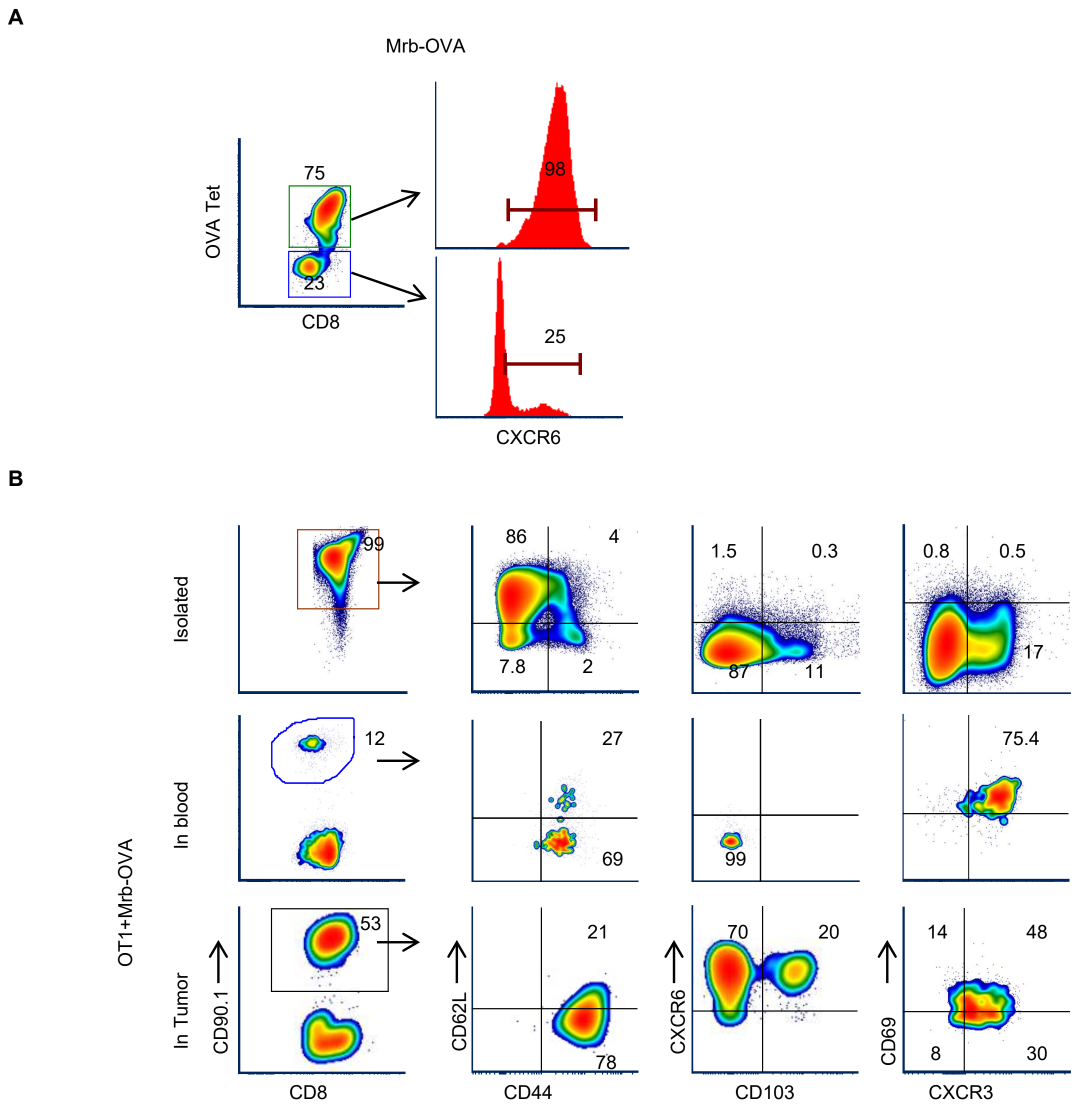

Supplementary figure 3. CXCR6 marks tumor specific resident memory cells. **(A)** CXCR6 expression in tumor specific (OVA tetramer pos) and non-tumor (OVA tetramer neg) CD8+ T cells in tumors of mice treated with Maraba-OVA vaccination (Mrb-OVA). **(B)** phenotype of OT1 T cells that were freshly isolated (top panel) from the donor mice or 7 days after vaccination in blood (middle panel) and in endpoint tumors (bottom panel) of mice treated with adoptive cell transfer of OT1 T cells and Maraba-OVA vaccination (OT1+Mrb-OVA).

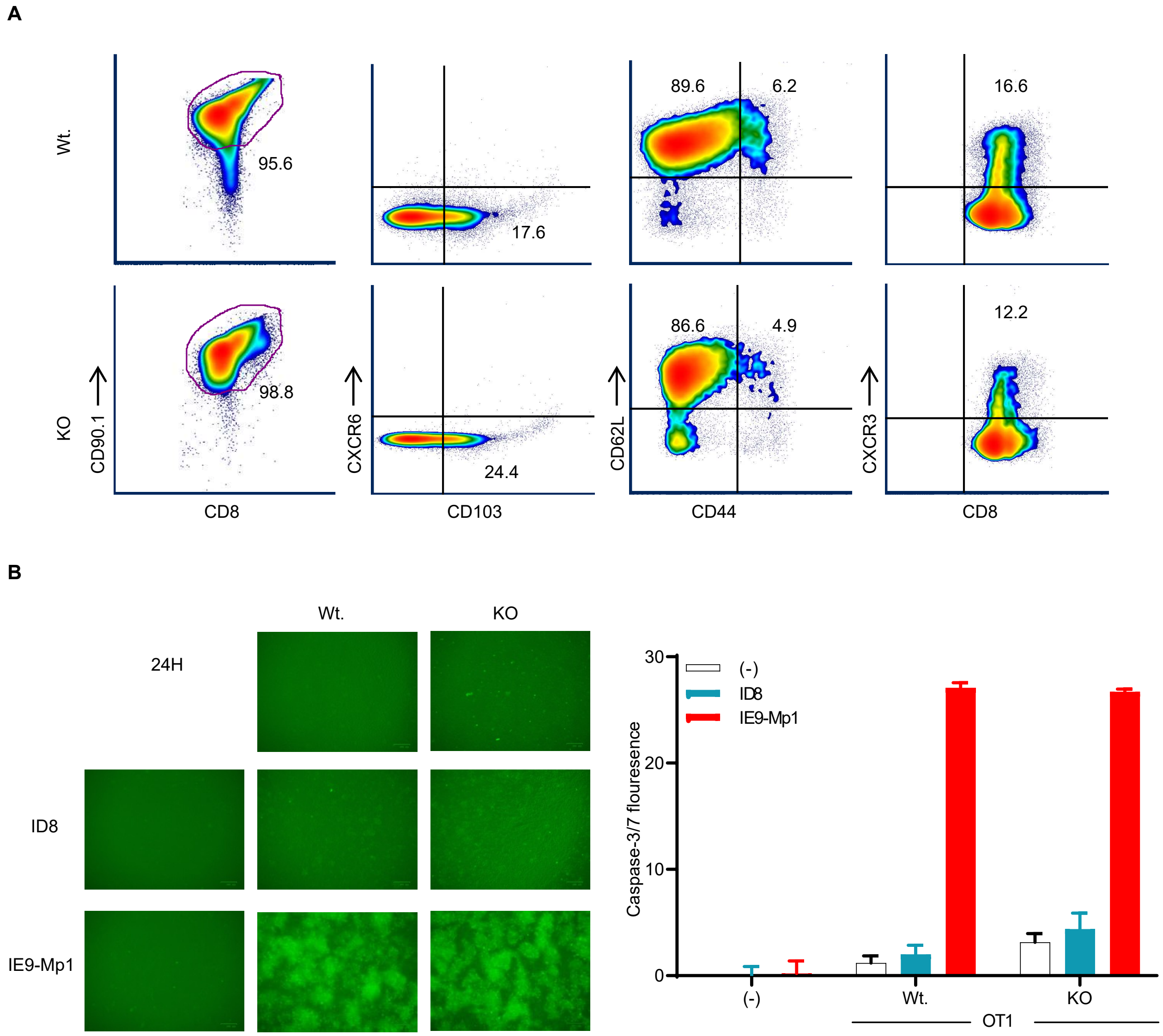

Supplementary figure 4. CXCR6 knockout did not affect phenotype or killing function of OT1 T cells. (A) Phenotype analysis of wild (Wt.) or CXCR6KO (KO) OT1 that are freshly isolated from mice for transfer into recipient mice. **(B)** Ability to Wt. or KO OT1 cells to induce activated CASPASE-3/7 in OVA expressing IE9-Mp1 or non-expressing ID8 control in 24-hour co-culture at effector: target ratio 10:1. Left panel shows fluorescence images taken on and right bar graphs shows mean of 3 replicate fluorescence values of various single or co cultures as measured on Biotek Synergy HT microplate plate reader at emission 528nM

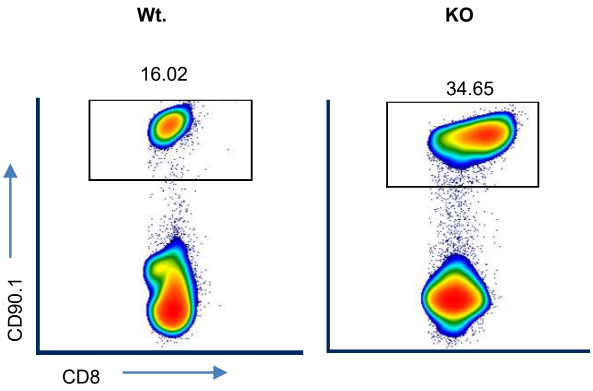

Supplementary figure 5. CXCR6KO OT1 accumulate more than wt. OT1 cells in spleens of B6 recipient mice. FACS dot blots of % CD90.1⁺ T cells in spleen of **o**ne representative B6 recipient mice that received ACT of either Wt. or KO OT1 cells +Mrb-OVA vaccine.
